# An integrated SWOT-AHP strategic positioning framework for assessing sustainable forest wellness tourism development in mountain regions

**DOI:** 10.1038/s41598-026-60242-1

**Published:** 2026-06-29

**Authors:** Longlong Liu, Enyi Zhou, Mohammad Heydari

**Affiliations:** 1 School of Management, Xi’an University of Architecture and Technology, Xi’an, 710055 China; 2 School of Economics and Management, Shangluo University, Shangluo, 726000 China; 3 School of Applied Business, Unitec Institute of Technology, Te Pūkenga, Auckland, New Zealand

**Keywords:** Forest wellness tourism, Sustainable development, SWOT-AHP, Strategic planning, Mountain tourism, Emerging economies, China, Ecology, Ecology, Environmental sciences, Environmental social sciences

## Abstract

Forest wellness tourism is an emerging form of nature-based tourism that leverages forest ecosystem services for health promotion while contributing to regional sustainable development. Although the integration of Strengths–Weaknesses–Opportunities–Threats (SWOT) analysis and the Analytic Hierarchy Process (AHP) has been applied in tourism planning, their combination with quantitative strategic positioning remains underexplored in the context of forest wellness tourism development in emerging economy mountain regions. This study applies an integrated SWOT-AHP strategic positioning framework to assess the sustainable development of forest wellness tourism in Shangluo City, Qinling Mountains, China. Through multi-round Delphi consultation (n = 46 for factor identification; n = 28 for AHP pairwise comparisons), 18 factors were identified across four SWOT dimensions. AHP weighting revealed that the Strengths dimension received the highest overall weight (dimension weight = 0.3652), with ecological resource quality ranking as the highest-weighted individual factor (composite weight = 0.1175), followed by policy support frameworks (0.0985) and traditional wellness culture (0.0871). Infrastructure deficits constituted the most prominent weakness (composite weight = 0.0647, representing 53.96% of total Weaknesses weight). Four-quadrant strategic positioning yielded coordinates P(0.2454, 0.1706), an intensity coefficient ρ = 0.2989, and an azimuth angle θ = 34.82°, placing Shangluo in Quadrant I (Strengths–Opportunities) and suggesting a competitive strategy that leverages internal advantages to capitalise on external opportunities. Sensitivity analysis confirmed the robustness of this positioning across all tested scenarios (ρ ranging from 0.2632 to 0.3489; Quadrant I maintained throughout). All findings should be interpreted as expert-perceived strategic priorities derived from structured expert judgment, rather than as objectively verified empirical outcomes of actual tourism demand, ecological measurement, or economic impact assessment.The findings provide case-based insights into how ecological resource endowment and institutional capacity are perceived by domain experts to jointly shape tourism development priorities in China’s western mountain regions, and illustrate one contextually grounded approach to reconciling economic development with ecological conservation in comparable mountain destination contexts.

## Introduction

The global wellness tourism sector reached an estimated $639 billion in 2023, with projected annual growth rates of 16.6% through 2030, substantially exceeding conventional tourism growth of approximately 5.8%^1^. This expansion reflects fundamental societal shifts, including intensifying urbanisation pressures, escalating chronic disease burdens—with non-communicable diseases accounting for 71% of global deaths—and growing health consciousness among populations with rising disposable incomes^2^. Within this broader wellness tourism ecosystem, forest wellness tourism has emerged as a distinctive paradigm that deliberately leverages forest ecosystem services to promote holistic health outcomes encompassing physiological, psychological, and social dimensions^3^.

The conceptual origins of forest wellness tourism are most commonly traced to the Japanese practice of shinrin-yoku (forest bathing), developed in the 1980s as a form of preventive health intervention integrating traditional nature-based healing with contemporary medicine^4^. A substantial body of clinical research has since established measurable health benefits associated with forest exposure. Park et al.^5^ demonstrated that forest environments produced significant reductions in salivary cortisol (13.4%), systolic blood pressure (1.9%), and pulse rate (6.0%) across 24 experimental sites in Japan. Li et al.^6^ established that forest exposure enhances natural killer (NK) cell activity—a key component of innate immune defence—with effects persisting up to 30 days post-exposure. Meta-analytical synthesis by Kotera, Richardson, and Sheffield^7^, aggregating 28 controlled studies (n = 2725), confirmed moderate-to-large effect sizes for anxiety reduction (Cohen’s d = 0.61), depression alleviation (d = 0.54), and mood improvement (d = 0.71). More recent post-pandemic studies have documented heightened mental health benefits of forest-based interventions, particularly among populations experiencing prolonged urban confinement^8,9^.

The development of forest wellness tourism as an organised sector varies considerably across national contexts, offering instructive comparisons for emerging programmes. South Korea established a national Forest Therapy programme in 2012 under the Korea Forest Service, which by 2023 operated over 40 designated healing forests and trained more than 1000 certified forest therapy instructors; clinical evaluations have documented significant reductions in stress biomarkers and improvements in mood states among participants^10–12^. In Central Europe, Germany has a century-long tradition of Kurwald (healing forest) designations linked to the Kneipp hydrotherapy tradition, with certified therapeutic forests integrated into health insurance reimbursement systems in several regions^13^. Finland has adopted nature-based wellness as a strategic tourism differentiation tool, leveraging extensive boreal forests (73% land coverage) and a well-established research infrastructure connecting forest exposure to stress recovery and attention restoration^14^. These international experiences share common themes—the importance of scientific evidence to underpin health claims, the role of institutional frameworks in standardising service quality, and the challenge of balancing visitor access with ecological integrity—that are directly relevant to emerging programmes in China.

In China, the convergence of rapid urbanisation—with over 900 million urban residents facing environmental and psychological stressors^15^—and strong cultural resonance with Traditional Chinese Medicine (TCM) principles has created substantial demand for nature-based healing experiences^16^. The national government has elevated forest wellness tourism to a strategic priority: the “Healthy China 2030” blueprint identifies forest-based therapies as cornerstone strategies for transitioning toward preventive health systems^17^, and the National Forestry and Grassland Administration (NFGA) established quantitative targets of 500 national-level forest wellness bases by 2025, with projected annual output exceeding ¥500 billion ($70 billion)^18^. By 2024, China had established over 1400 forest wellness bases generating revenues approaching $10.2 billion annually^19^. Post-pandemic market analyses indicate structural demand shifts, with wellness tourism recovering substantially faster than conventional tourism segments, and forest-based experiences commanding 23–31% price premiums in comparable domestic markets^20,21^.

Strategically planning for forest wellness tourism development requires systematic assessment of multiple internal and external factors—that is, factors that significantly influence strategic development decisions and whose quantified priority weights reflect their relative influence on overall development outcomes^22^. Strengths–Weaknesses–Opportunities–Threats (SWOT) analysis provides a widely adopted conceptual framework for such assessment but suffers from well-documented limitations, including subjectivity in factor identification, implicit equal-weighting assumptions, predominantly qualitative emphasis, and ambiguity in translating analysis into implementation priorities^23^. Beyond its methodological application, this study advances two theoretically grounded arguments. First, the simultaneous identification of ecological resources as both the dominant competitive asset and the primary source of ecological threat—a tension here termed the “conservation-exploitation paradox”—offers a transferable conceptual insight for nature-dependent wellness tourism planning that extends beyond the Shangluo case. Second, the prominent positioning of government policy factors across both internal Strengths (S_3_) and external Opportunities (O_1_) dimensions provides empirical illustration of how institutional capacity mediates the conversion of natural resource endowments into competitive advantage, a relationship theorised in destination competitiveness models but rarely examined through quantitative expert-weighting approaches in wellness tourism contexts. These contributions are conceptually distinct from the procedural application of the SWOT-AHP framework and are elaborated in Section “Theoretical implications”.Integration with the Analytic Hierarchy Process (AHP), which enables structured pairwise comparisons and mathematical derivation of priority weights with built-in consistency checks^24^, addresses several of these limitations by introducing quantitative rigour to what is otherwise a qualitative exercise.

The SWOT-AHP hybrid framework was pioneered by Kurttila et al.^25^ in the context of forest management and certification in Finland. Pesonen et al.^26^ subsequently applied the A’WOT (AHP-SWOT) method to evaluate resource management strategies at the Finnish Forest and Park Service, demonstrating that the hybrid approach yielded more discriminating priority rankings than conventional SWOT alone. Since then, the method has been applied across diverse planning contexts: Kajanus et al.^27^ provided a comprehensive methodological synthesis of multi-criteria decision support SWOT applications in natural resource management; Ghorbani et al.^28^ employed SWOT-AHP to develop ecotourism strategies for wetland areas in Iran; and Akbulak and Cengiz^29^ applied the A’WOT hybrid method to national park tourism planning in Turkey. In tourism specifically, applications have spanned coastal tourism sustainable development, cultural heritage tourism strategy, and ecotourism planning in diverse geographical settings^30^.

Across these prior SWOT-AHP tourism studies, several recurring factor patterns emerge that inform theoretical expectations for the present study. Infrastructure adequacy—particularly transportation accessibility and accommodation capacity—consistently appears as a critical weakness or bottleneck factor in emerging economy tourism contexts. Government policy and institutional support frequently emerge as high-weighted strengths or opportunities, especially in studies conducted in developmental state contexts where public investment catalyses private sector participation^31^. Natural resource endowments typically appear as strengths but often receive lower AHP priority weights than institutional or market-access factors in studies with well-resourced destinations, suggesting that resource possession alone is insufficient without complementary enabling conditions^32^. Ecological fragility and environmental degradation risks appear consistently as threats, though their relative weights vary substantially depending on governance capacity and regulatory enforcement. These patterns generate a theoretical expectation that in the present case—a resource-rich but infrastructure-constrained mountain region in western China—ecological resource factors may provide the competitive foundation, while institutional and infrastructure factors may determine the pace and quality of development.

However, most existing SWOT-AHP applications in tourism remain limited to factor ranking and do not incorporate quantitative strategic positioning analysis. That is, while they determine which factors matter most, they do not calculate the overall strategic direction, intensity, or quadrant placement that would guide implementation. This study extends the existing approach by integrating four-quadrant coordinate analysis with calculated intensity coefficients (ρ) and azimuth angles (θ), following the positioning logic developed by Weihrich^33^, to provide additional precision in translating SWOT-AHP results into strategic directional guidance. It should be noted that this integration is not novel to this study; our contribution lies in the systematic application of this established framework to the specific context of forest wellness tourism in western China, with enhanced procedural transparency—including detailed Delphi protocols, operational factor definitions, inter-group comparisons, and sensitivity analysis—that addresses methodological concerns raised in the extant literature^23^.

The challenges facing forest wellness tourism development in mountain regions are situated within broader debates on sustainable mountain tourism. Mountain regions worldwide face dual constraints: persistent economic underdevelopment and ecological fragility that limits conventional development pathways^34,35^. Infrastructure consistently emerges as a binding constraint in emerging economy mountain contexts, with construction costs typically exceeding lowland equivalents by 30–50%, and temporal lags between policy commitment and physical delivery creating persistent implementation gaps^36^. Governance quality significantly influences development outcomes; multi-level governance analyses in China have revealed persistent policy-practice gaps attributable to institutional fragmentation, inadequate enforcement, and misaligned incentives across administrative levels^37^. More recent research has highlighted the amplified importance of digital connectivity infrastructure in post-pandemic tourism, with 5G coverage and IoT integration becoming increasingly important for visitor experience management and environmental monitoring^38,39^. Successful cases of mountain tourism development demonstrate the importance of integrated planning frameworks, participatory stakeholder engagement, and adaptive management approaches that balance development urgency with conservation imperatives^40,41^.

Shangluo City, situated in the Qinling Mountains of Shaanxi Province, exemplifies the development paradoxes common to emerging economy mountain regions. Despite exceptional natural endowments—including 69.56% forest coverage, favourable summer climate conditions, rich biodiversity (1728 documented plant species), and strategic proximity to Xi’an’s 13 million residents (approximately 150 km)—tourism development remains nascent^42^. The region faces a set of interconnected development challenges—namely, infrastructure deficits limiting visitor accessibility, service standardisation gaps reducing repeat visitation, limited brand recognition among target consumer segments, and inter-agency coordination difficulties fragmenting planning efforts—that collectively constrain the translation of resource endowments into competitive tourism products^42^.

This study applies an integrated SWOT-AHP strategic positioning framework to the case of forest wellness tourism in Shangluo to address three research questions.

RQ1 asks: What are the key internal and external factors affecting forest wellness tourism development in Shangluo, and how are they distributed across the four SWOT dimensions?

RQ2 asks: What is the relative importance of these factors as determined through AHP weighting, and how should they be prioritised to inform resource allocation decisions?

RQ3 asks: What overall strategic orientation does the integrated analysis suggest, and how robust is this positioning to variations in input assumptions?

## Methodology

### Study area characteristics

Shangluo City (108°34′20″–111°1′25″E, 33°2′30″–34°24′40″N) is located in the southeastern region of Shaanxi Province, China, on the southern flank of the Qinling Mountains. The municipality administers one district (Shangzhou) and six counties (Luonan, Danfeng, Shangnan, Shanyang, Zhen’an, and Zhashui), encompassing a total area of approximately 19,292 km^2^. As of 2022, Shangluo had a permanent resident population of 2.11 million and a GDP of 90.1 billion yuan (approximately $12.7 billion USD)^43^.

The study area’s relevance to forest wellness tourism derives from several distinctive characteristics. Forest coverage reaches 69.56%, the highest among all prefecture-level cities in Shaanxi Province and substantially above the national average of 24.02%^44^. The Qinling Mountains constitute a critical biogeographic boundary between the Palearctic and Oriental realms, supporting exceptional biodiversity including over 3400 seed plant species and 500 + vertebrate species, with notable populations of giant panda (Ailuropoda melanoleuca), golden snub-nosed monkey (Rhinopithecus roxellana), crested ibis (Nipponia nippon), and golden takin (Budorcas taxicolor bedfordi)^45^. Previous environmental monitoring studies have documented negative air ion concentrations in Shangluo’s primary forest areas ranging from 3000 to 12,000 ions/cm^3^, substantially exceeding the therapeutic threshold of 1000 ions/cm^3^ proposed in forest medicine literature^46^. The region’s climate is classified as warm temperate to subtropical transitional, with annual mean temperatures of 11–14 °C and a summer mean of 22–26 °C, placing it within the “comfortable” range on the Tourism Climate Index ^47^.

From a market access perspective, Shangluo is located approximately 110 km east of Xi’an (population 13 million), connected by the G40 Shanghai–Xi’an Expressway (approximately 1.5 h driving time) and the existing Xi’an–Ankang railway (approximately 2.5 h)^48^. The Xikang High-Speed Railway, currently under construction with an expected completion date of 2026, will reduce travel time to approximately 40 min^49^, fundamentally altering the region’s accessibility profile.

Field reconnaissance was conducted across all seven administrative divisions between March and June 2023, encompassing visits to 23 existing forest parks, nature reserves, and scenic areas. These site visits informed the initial identification of candidate strategic factors and provided contextual grounding for the subsequent Delphi and AHP processes.

### Research design overview

**Fig. 1:**
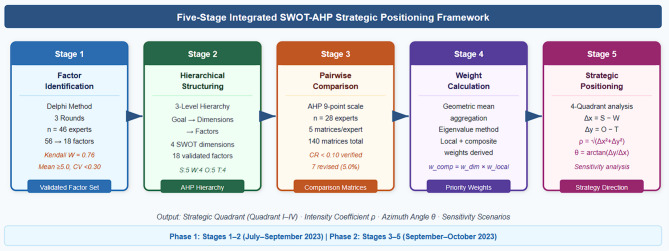
Five-stage integrated SWOT-AHP-strategic positioning analytical framework.

This study employs a sequential mixed-methods design integrating three established analytical techniques—SWOT analysis, the Delphi method, and the Analytic Hierarchy Process (AHP)—into a structured strategic assessment framework (Fig. 1). While each technique is well-established independently, their integration addresses specific limitations that arise when any single method is applied alone^25^. Standard SWOT analysis produces unranked lists of strategic factors without indicating their relative importance. The Delphi method addresses factor validation through structured expert consensus but does not inherently quantify relative importance. AHP provides ratio-scale priority weights but requires a pre-defined factor set as input. By chaining these methods sequentially—Delphi for factor identification and validation, AHP for quantitative prioritisation, and SWOT as the organising strategic framework—the integrated approach compensates for the limitations of each individual method.

The research proceeded in two sequential phases. Phase 1 (Delphi consultation, July–September 2023) established the validated factor set through three iterative rounds of expert consultation. Phase 2 (AHP weighting, September–October 2023) quantified relative factor importance through pairwise comparison judgments. The two phases employed overlapping but non-identical expert panels, as detailed in Section “Expert panel composition”.

The five stages of the framework operate as follows. Stage 1 (Factor Identification) employs multi-round Delphi consultation with 46 experts to refine factors from an initial pool of 56 candidates to a final validated set of 18, using dual criteria of mean importance rating ≥ 5.0 and coefficient of variation < 0.30^50^. Stages 2–3 (Hierarchical Structuring and Pairwise Comparison) have an expert subset (n = 28) complete pairwise comparisons using Saaty’s 9-point scale, with consistency verification (CR < 0.10)^51^. Stage 4 (Weight Calculation) derives priority weights using geometric mean aggregation, with composite weights integrating hierarchical levels. Stage 5 (Strategic Positioning) determines the strategic centre P(x, y) where x = S − W and y = O − T, the intensity coefficient ρ = √(Δx^2^ + Δy^2^), and the azimuth angle θ = arctan(Δy/Δx).

#### Delphi method

The Delphi process was designed following procedural guidelines recommended for quality assurance in Delphi studies^50^. Three rounds were conducted between July and September 2023.

*Round* 1 (*July* 2023): Experts received a structured questionnaire containing 56 candidate strategic factors organised into four SWOT dimensions (Strengths: 16; Weaknesses: 14; Opportunities: 14; Threats: 12). Each factor was accompanied by a brief description providing operational context. Experts rated each factor’s relevance and importance on a 9-point scale (1 = not important, 9 = extremely important) and were invited to suggest additional factors through open-ended comments. The retention threshold was set at a mean score ≥ 5.0 and a coefficient of variation (CV) ≤ 0.30. Round 1 resulted in the retention of 38 factors, elimination of 8, and flagging of 10 for re-evaluation.

*Round* 2 (*August* 2023): Experts received the revised factor list (48 factors: 38 retained + 10 flagged) along with anonymised summary statistics from Round 1. Experts re-rated flagged factors and were invited to revise ratings for retained factors. Several overlapping factors were consolidated (e.g., three separate transportation-related weakness factors were merged into a single “infrastructure deficits” factor). Round 2 resulted in a refined set of 22 factors (Strengths: 7; Weaknesses: 5; Opportunities: 5; Threats: 5).

*Round* 3 (*September* 2023): A final confirmation round presented the 22 surviving factors with Round 2 statistics. Factors receiving > 80% confirmation were retained; those receiving 60–80% were discussed in a moderated online session; those receiving < 60% were eliminated. This process yielded the final validated factor set of 18 factors (Strengths: 5; Weaknesses: 4; Opportunities: 5; Threats: 4). The reduction to four weaknesses in the final set reflects the Round 2 consolidation process, during which a previously merged brand-awareness indicator was re-established as a discrete factor (W_4_: Limited brand recognition) following expert feedback that it captured a qualitatively distinct constraint from service standardisation. Conversely, a seasonality-related threat factor present in Round 2 was eliminated in Round 3 on the grounds that it partially overlapped with market uncertainty (T_3_) and failed to reach the 80% confirmation threshold. Consensus was assessed using Kendall’s coefficient of concordance (W), which reached 0.76 in Round 3 (*p* < 0.001), indicating substantial agreement.

#### Analytic hierarchy process (AHP)

AHP was implemented following the standard procedure described by Saaty^24^, comprising hierarchy construction, pairwise comparison, priority calculation, and consistency verification, as expounded in detail by Forman and Gass^51^.

*Hierarchy structure*: A three-level hierarchy was constructed: Level 1 (goal) represents the strategic assessment of forest wellness tourism development; Level 2 (dimensions) comprises the four SWOT dimensions; Level 3 (factors) includes the 18 validated factors distributed across the four dimensions (Strengths: 5; Weaknesses: 4; Opportunities: 5; Threats: 4).

*Pairwise comparison*: Experts completed five comparison matrices—one 4 × 4 matrix comparing the four SWOT dimensions, and four within-dimension matrices (5 × 5 for Strengths, 4 × 4 for Weaknesses, 5 × 5 for Opportunities, 4 × 4 for Threats)—using Saaty’s standard 1–9 scale. To reduce respondent burden, experts completed comparisons electronically using a custom-designed survey instrument that presented one paired comparison at a time with visual scale anchors.

*Priority calculation*: Individual comparison matrices were aggregated using the geometric mean method, which is the only aggregation method that preserves the reciprocal property of pairwise comparison matrices. For a comparison matrix A of order n, the priority vector w is the principal eigenvector satisfying:

1 $$A \ w = \lambda_{\max } \ w$$

where:

- A is the pairwise comparison matrix,
- w is the priority (eigenvector),
- λ_max_ is the largest eigenvalue of *A*.

*Consistency verification*: Consistency was assessed using the Consistency Ratio (CR):

2 $$CR = \frac {CI} {RI}$$

with:

3 $$CI = \frac{\lambda_{max}-n}{n-1}$$

where CI = (λmax − n)/(n − 1), and RI is the average consistency index of randomly generated reciprocal matrices of the same order (n = 4: RI = 0.90; n = 5: RI = 1.12)^52^. Matrices with CR > 0.10 were returned to respondents for revision; this occurred for 7 of the initial 140 matrices (5.0%), all of which achieved acceptable consistency after revision.

*Composite weight calculation*: Composite (global) weights were calculated as the product of dimension-level and factor-level local weights:

4 $$w_{{{\mathrm{composite}},{\mathrm{i}}}} = w_{{{\mathrm{dimension}},{\mathrm{j}}}} \times w_{{{\mathrm{local}},{\mathrm{i}}}}$$

where *w*_dimension,j_ is the weight of the SWOT dimension to which factor *i* belongs, and *w*_local,i_ is the factor’s weight within that dimension.

#### Strategic quadrant positioning

The strategic position was determined by calculating aggregate strength–weakness and opportunity–threat coordinates:

5 $$\Delta {X} = \sum_i {W_{S,i}} - \sum_j {W_{W,j}}$$

6 $$\Delta {Y} = \sum_k {W_{O,k}} - \sum_l {W_{T,l}}$$

where:

- *w*_S,i_: composite weights of Strength factors,
- *w*_W,j_: composite weights of Weakness factors,
- *w*_O,k_: composite weights of Opportunity factors,
- *w*_T,l_: composite weights of Threat factors.

and  are the composite weights of individual Strength, Weakness, Opportunity, and Threat factors, respectively.

The strategic azimuth angle (θ) and intensity coefficient (ρ) were calculated as:

7 $$\theta = {\mathrm{arctan}}\left( {\Delta {\mathrm{y}}/\Delta {\mathrm{x}}} \right)$$

8 $$\rho = \surd \left( {\Delta {\mathrm{x}}^{{2}} + \Delta {\mathrm{y}}^{{2}} } \right)$$

The azimuth angle indicates strategic direction (0–90° = Quadrant I/SO growth strategy; 90–180° = Quadrant II/WO adjustment strategy; 180–270° = Quadrant III/WT survival strategy; 270–360° = Quadrant IV/ST diversification strategy), while the intensity coefficient indicates the magnitude of the strategic signal on the positioning plane.

### Expert panel composition

Expert selection followed a criterion-based purposive sampling strategy^53^ designed to ensure disciplinary breadth, practical relevance, and regional knowledge. Candidates were required to satisfy at least one of the following eligibility criteria: (a) academic researchers with peer-reviewed publications in forest ecology, tourism management, health geography, or Traditional Chinese Medicine; (b) government officials at municipal or county level with direct portfolio responsibility for tourism planning, forestry management, or economic development in Shangluo; (c) tourism industry practitioners with ≥ 5 years of operational experience in nature-based tourism enterprises in the Qinling region; and (d) health and wellness professionals (physicians, traditional medicine practitioners, public health specialists) with knowledge of forest-based health interventions. Disciplinary diversity was further ensured by setting target quotas for each professional category prior to recruitment.

Recruitment proceeded through institutional contacts established during preliminary fieldwork (March–June 2023), supplemented by snowball referrals to identify candidates not reachable through institutional channels. Initial invitations were sent to 62 candidates; 46 agreed to participate in Phase 1 (Delphi), yielding a response rate of 74.2%. Of these, 28 continued to Phase 2 (AHP), yielding a Phase 2 retention rate of 60.9%. The reduction from 46 to 28 participants between phases was attributable to time constraints (n = 12) and non-response to the AHP survey instrument (n = 6); no systematic demographic differences were observed between continuing and non-continuing participants (chi-square tests for professional category, *p* = 0.42; years of experience, *p* = 0.61).

The final panel composition was as follows. Phase 1 (Delphi, n = 46): academic researchers 15 (32.6%), government officials 12 (26.1%), industry practitioners 11 (23.9%), health professionals 8 (17.4%). Phase 2 (AHP, n = 28): academic researchers 10 (35.7%), government officials 7 (25.0%), industry practitioners 6 (21.4%), health professionals 5 (17.9%). Mean professional experience across both phases was 14.3 years (SD = 6.8, range 5–32 years). All experts were briefed on the study objectives and provided written informed consent prior to participation. The research protocol was approved by the Ethics Committee of Xi’an University of Architecture and Technology (Protocol #2023-HM-089).

### Factor identification and operational definitions

The initial pool of 56 candidate factors was compiled through three complementary sources: (a) systematic review of 47 peer-reviewed publications on SWOT analysis in nature-based and forest tourism contexts (2015–2023); (b) analysis of 12 government policy documents related to forest wellness tourism development in Shaanxi Province and nationally; and (c) semi-structured interviews with 15 key informants during preliminary fieldwork (March–June 2023). Following the three-round Delphi process, 18 factors were validated for inclusion in the AHP analysis. Table 1 presents these validated factors with operational definitions and expert rating statistics from the final Delphi round.

**Table 1:** Validated SWOT factors with operational definitions and expert rating statistics (final Delphi round).

Code	Factor name	Operational definition	Mean	SD	CV
Strengths (S)					
S_1_	Ecological resource quality	Overall quality of forest ecosystems assessed by biodiversity indices, vegetation coverage, air quality parameters (negative air ion concentration, phytoncide levels), and water quality. Encompasses objective ecological condition and suitability for wellness-oriented tourism activities	6.795	1.342	0.198
S_2_	Traditional wellness culture	Accumulated body of traditional health-related knowledge and practices associated with the Qinling region, including TCM herbal traditions, Taoist health cultivation practices, local folk medicine knowledge, and documented historical use of forest environments for therapeutic purposes	6.138	1.521	0.248
S_3_	Government policy attention	Degree to which municipal and county-level governments have incorporated forest wellness tourism into official development plans, allocated dedicated budgets, established coordinating institutions, and issued supportive policy documents. Distinct from O_1_, which refers to higher-level policy frameworks	7.232	1.187	0.164
S_4_	Geographic location and accessibility	Shangluo’s spatial relationship to major source markets (primarily Xi’an metropolitan area), measured by travel time, transportation mode availability, and route connectivity, including current accessibility and confirmed near-term improvements (Xikang High-Speed Railway)	6.517	1.408	0.216
S_5_	Climate comfort	Suitability of the local climate for outdoor wellness activities across seasons, assessed through the Tourism Climate Index, with particular emphasis on summer comfort advantage relative to source market cities	7.325	1.095	0.149
Weaknesses (W)					
W_1_	Infrastructure deficits	Gaps in physical infrastructure required for wellness tourism operations, including intra-regional transportation networks (last-mile connectivity to forest sites), accommodation capacity and quality, medical/emergency facilities, digital connectivity (mobile and broadband coverage in forest areas), and basic utilities at potential wellness sites	6.888	1.264	0.184
W_2_	Professional talent shortage	Insufficient availability of personnel with specialised qualifications relevant to forest wellness tourism, including certified forest therapy guides, wellness programme designers, TCM practitioners willing to work in tourism contexts, bilingual hospitality staff, and tourism management professionals with wellness domain knowledge	5.342	1.587	0.297
W_3_	Low service standardisation	Absence of established quality standards, service protocols, and certification systems for forest wellness tourism products and services in the region, including lack of standardised wellness activity protocols, inconsistent service quality across providers, and absence of outcome measurement systems	5.579	1.456	0.261
W_4_	Limited brand recognition	Low awareness of Shangluo as a forest wellness destination among target consumer segments in key source markets, including both general destination awareness and specific association with wellness/health tourism attributes, assessed relative to competing destinations in the Qinling region	5.183	1.502	0.290
Opportunities (O)					
O_1_	National/provincial policy support	Higher-level (national and provincial) government policy frameworks creating enabling conditions for forest wellness tourism development, including the Healthy China 2030 initiative, NFGA National Forest Wellness Base certification programme, Shaanxi Provincial ecotourism development plans, and associated fiscal incentives. Distinct from S_3_, which refers to local government initiative	6.875	1.229	0.179
O_2_	Xi’an market access	Potential demand for forest wellness tourism from the Xi’an metropolitan area (population ~ 13 million), including growth in disposable income, increasing health consciousness, weekend/short-break travel patterns, and the demand–supply gap for quality nature-based wellness experiences within a 2-h travel radius	6.987	1.153	0.165
O_3_	Wellness industry growth	Macro-level growth trends in China’s wellness and health tourism industry, including market size expansion, product diversification, increasing consumer willingness to pay for wellness experiences, and post-pandemic acceleration of health-oriented leisure demand	5.979	1.384	0.231
O_4_	Public health awareness	Rising public awareness of and demand for preventive health practices, stress management, and nature-based health interventions among urban Chinese populations, including the growing scientific evidence base for forest therapy benefits and its dissemination through popular media	5.408	1.519	0.281
O_5_	Technology integration potential	Opportunities to enhance forest wellness tourism experiences through technology applications, including wearable health monitoring devices, AI-personalised wellness programmes, VR/AR-enhanced nature interpretation, smart trail management systems, and digital marketing/booking platforms	5.125	1.498	0.292
Threats (T)					
T_1_	Ecological fragility	Vulnerability of Shangluo’s forest ecosystems to degradation from tourism pressure, climate change, and other anthropogenic stressors, including risks of biodiversity loss, habitat fragmentation, soil erosion on mountain trails, water pollution from tourism facilities, and noise disturbance to wildlife	5.246	1.541	0.294
T_2_	Regional competition	Competitive pressure from other destinations in the Qinling region and broader western China developing similar forest wellness tourism products, including Foping, Liuba, and Taibai counties in Shaanxi; Kangxian in Gansu; and established forest wellness bases in Sichuan and Yunnan	6.442	1.371	0.213
T_3_	Market uncertainty	Risks associated with demand volatility, including sensitivity to economic cycles, potential for public health emergencies to disrupt travel, uncertainty about the long-term trajectory of wellness tourism demand, and the possibility that forest wellness may prove a transient rather than sustained market segment	5.158	1.476	0.286
T_4_	Financing constraints	Difficulties in securing adequate investment capital for forest wellness tourism development, arising from long payback periods, risk aversion among private investors, limited local government fiscal capacity, and competition for investment capital from other development priorities	5.046	1.509	0.299

Rating scale 1–9. All factors meet dual validation criteria: Mean ≥ 5.0 and CV < 0.30. SD = standard deviation; CV = coefficient of variation.

### Data collection procedures

*Phase* 1 (*Delphi*): All three Delphi rounds were administered through an online survey platform, with a response window of 14 days per round. Between rounds, the research team compiled descriptive statistics (mean, median, standard deviation, interquartile range, coefficient of variation) for each factor and prepared anonymised feedback summaries. Experts who had not responded within 10 days received a single reminder. The inter-round interval was approximately three weeks. During recruitment, initial response rates from government officials were lower than anticipated (62% vs. target 80%), necessitating follow-up contacts and an extended collection period; these experiences informed the final response rate of 74.2%.

*Phase* 2 (*AHP*): AHP comparison matrices were administered through a structured Excel-based instrument distributed by email. Each expert received a workbook containing five comparison matrix worksheets with embedded instructions and visual examples of the 1–9 scale interpretation. Some experts requested clarification on distinguishing between ratings of 7 and 9 on the pairwise scale, which the research team addressed through supplementary examples distributed individually. Completed instruments were returned within a 21-day window (September 15–October 5, 2023). Each returned matrix was checked for consistency (CR ≤ 0.10) within 48 h; experts whose matrices exceeded the consistency threshold were contacted and asked to revise their judgments with specific guidance on which comparisons were most inconsistent.

### Sensitivity analysis

To assess the robustness of the strategic positioning results to variations in dimension-level weights, a systematic sensitivity analysis was conducted. The base-case weights for each of the four SWOT dimensions were individually perturbed by ± 10% and ± 20%, with the remaining three dimensions’ weights rescaled proportionally to maintain a sum of 1.0. This produced 16 scenarios (4 dimensions × 4 perturbation levels). For each scenario, composite weights were recalculated, and the strategic quadrant coordinates (Δx, Δy), azimuth angle (θ), and intensity coefficient (ρ) were recomputed to determine whether the perturbation altered the strategic quadrant assignment. Four scenarios that produced negligible changes were combined, yielding 12 distinct reported scenarios.

## Results

### Delphi consensus outcomes

The three-round Delphi process progressively narrowed the factor set from 56 initial candidates to 18 validated factors. Consensus, as measured by Kendall’s coefficient of concordance (W), improved across rounds: Round 1 W = 0.52, Round 2 W = 0.67, Round 3 W = 0.76 (all *p* < 0.001), indicating progressive convergence toward agreement consistent with effective feedback mechanisms.

The factor attrition pattern was as follows. From the initial 56 factors, Round 1 eliminated 8 factors that failed both retention criteria; these included factors judged too generic (e.g., “beautiful scenery”), too narrow (e.g., “specific rare plant species”), or redundant. Round 2 reduced the set to 22 by eliminating or consolidating 26 factors—notably, three separate transportation-related factors were merged into the single “infrastructure deficits” factor, and a previously separate brand-awareness indicator was retained as W_4_ following expert feedback that it captured a qualitatively distinct dimension from service standardisation (W_3_). Round 3 eliminated 4 additional factors through the confirmation vote process, including a seasonality-related threat factor that overlapped substantially with market uncertainty (T_3_) and failed to reach the 80% confirmation threshold. The final validated factor set comprised 18 factors distributed as 5 Strengths, 4 Weaknesses, 5 Opportunities, and 4 Threats.

### AHP weight results

The dimension-level comparison matrix yielded the following priority weights: Strengths (S) = 0.3652, Opportunities (O) = 0.3428, Threats (T) = 0.1722, Weaknesses (W) = 0.1198. The consistency ratio for the dimension-level matrix was CR = 0.032, well within the acceptable threshold of 0.10. This weight distribution indicates that the expert panel collectively judged Shangluo’s internal strengths and external opportunities as substantially more influential than its weaknesses and threats, providing the first indication of a favourable (Quadrant I) strategic position. The complete dimension-level and factor-level weights are presented in Table 2.

**Table 2:** AHP weight results: dimension-level and factor-level weights.

Dimension	Dimension Weight	Factor	Local Weight	Composite Weight	Global Rank
Strengths (S) 0.3652 CR = 0.032	0.3652	S_1_ Ecological resource quality	0.3217	0.1175	1
		S_2_ Traditional wellness culture	0.2385	0.0871	3
		S_3_ Government policy attention	0.1893	0.0691	6
		S_4_ Geographic location	0.1462	0.0534	9
		S_5_ Climate comfort	0.1043	0.0381	13
Weaknesses (W) 0.1198 CR = 0.046	0.1198	W_1_ Infrastructure deficits	0.5396	0.0647	7
		W_2_ Professional talent shortage	0.2472	0.0296	16
		W_3_ Low service standardisation	0.1335	0.0160	17
		W_4_ Limited brand recognition	0.0797	0.0095	18
Opportunities (O) 0.3428 CR = 0.028	0.3428	O_1_ Policy support frameworks	0.2873	0.0985	2
		O_2_ Xi’an market access	0.2534	0.0869	4
		O_3_ Wellness industry growth	0.2106	0.0722	5
		O_4_ Public health awareness	0.1554	0.0533	10
		O_5_ Technology integration	0.0933	0.0320	14
Threats (T) 0.1722 CR = 0.038	0.1722	T_1_ Ecological fragility	0.3189	0.0549	8
		T_2_ Regional competition	0.2653	0.0457	11
		T_3_ Market uncertainty	0.2348	0.0404	12
		T_4_ Financing constraints	0.1810	0.0312	15

CR = Consistency Ratio (all below 0.10 threshold). “Dim. CR” rows report within-dimension consistency ratios. The dimension-level 4 × 4 matrix CR = 0.032. Composite Weight = Dimension Weight × Local Weight. Global Rank based on composite weight values descending.

The factor-level results reveal notable concentration patterns within dimensions. Within Strengths, ecological resource quality (S_1_, composite weight 0.1175) is the dominant factor overall, receiving approximately 1.35 times the weight of the second-ranked strength (traditional wellness culture, S_2_, 0.0871). Within Weaknesses, infrastructure deficits (W_1_) dominate substantially, accounting for 53.96% of the total Weaknesses weight—a concentration that reflects expert consensus on infrastructure as the binding internal constraint. Within Opportunities, the top two factors—policy support frameworks (O_1_, 0.0985) and Xi’an market access (O_2_, 0.0869)—together account for 54.07% of Opportunities weight, indicating that institutional enablers and market proximity are viewed as the primary external facilitators. Within Threats, ecological fragility (T_1_, 0.3189) and regional competition (T_2_, 0.2653) together account for 58.42% of Threats weight.

To examine whether weight assignments varied systematically across professional categories, Table 3 presents dimension-level weights disaggregated by expert group.

**Table 3:** Dimension-level weights by professional category (Phase 2 AHP panel, n = 28).

Expert group	n	Strengths	Weaknesses	Opportunities	Threats
Academic researchers	10	0.3421	0.1387	0.3198	0.1994
Government officials	7	0.4012	0.0876	0.3753	0.1359
Industry practitioners	6	0.3587	0.1342	0.3401	0.1670
Health professionals	5	0.3624	0.1195	0.3389	0.1792
**Aggregated (all experts)**	**28**	**0.3652**	**0.1198**	**0.3428**	**0.1722**

Group-level weights calculated by applying the geometric mean aggregation method within each professional category separately. The aggregated row reflects the full-panel geometric mean (not a simple average of group weights). All four professional groups independently place Shangluo in Quadrant I (positive Δx and Δy).

The inter-group comparison reveals two noteworthy patterns. First, government officials assigned the highest Strengths weight (0.4012) and the lowest Weaknesses weight (0.0876) and Threats weight (0.1359) among all groups, producing the most optimistic assessment profile. Academic researchers, by contrast, assigned the highest Weaknesses (0.1387) and Threats (0.1994) weights, yielding the most cautious assessment. This divergence is consistent with known tendencies for institutional stakeholders to emphasise positive dimensions and for academic evaluators to adopt more critical perspectives^54^. Second, despite these differences, all four professional groups placed Shangluo in the same strategic quadrant (Quadrant I, SO strategy), with positive Δx and Δy values in every case. This cross-group consistency in quadrant assignment strengthens confidence in the overall strategic positioning, even as the within-quadrant location varies across groups.

### Strategic positioning

Using the composite weights from Table 2 and Eqs. (4)–(7), the strategic coordinates were calculated as follows:

$$\Delta {\mathrm{x}} = \Sigma \left( {{\mathrm{wS}}} \right) - \Sigma \left( {{\mathrm{wW}}} \right) = 0.{3652} - 0.{1198} = 0.{2454}$$

$$\Delta {\mathrm{y}} = \Sigma \left( {{\mathrm{wO}}} \right) - \Sigma \left( {{\mathrm{wT}}} \right) = 0.{3428} - 0.{1722} = 0.{17}0{6}$$

$$\theta = {\mathrm{arctan}}\left( {0.{17}0{6}/0.{2454}} \right) = {34}.{82}^\circ$$

$$\rho = \surd \left( {0.{2454}^{2} + 0.{17}0{6}^{2} } \right) = 0.{2989}$$

**Fig. 2:**
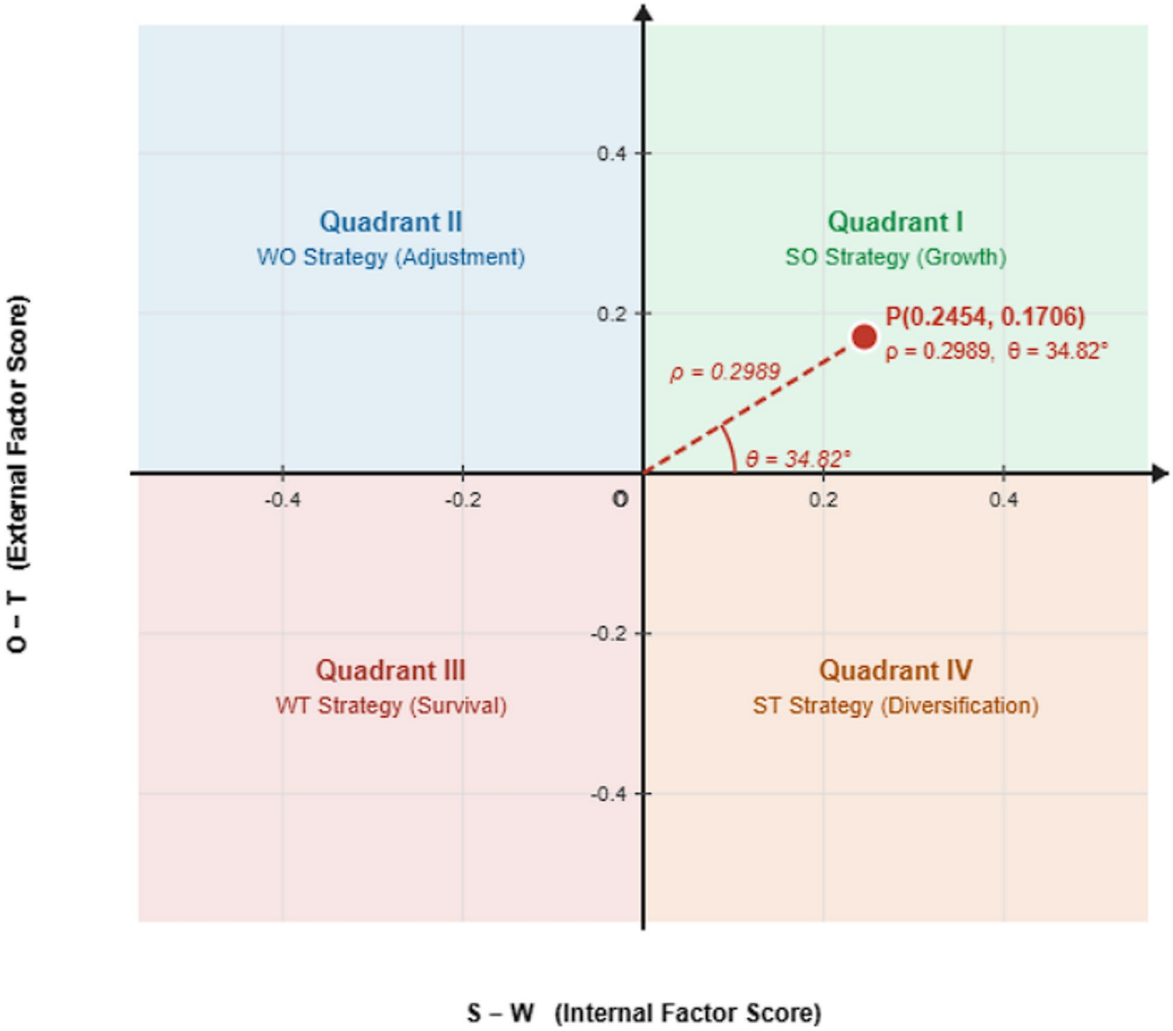
Four-quadrant strategic positioning matrix: forest wellness tourism development in Shangluo city.

Both coordinates are positive (Δx > 0, Δy > 0), placing Shangluo firmly in Quadrant I (SO strategy zone) of the strategic positioning matrix (Fig. 2). The azimuth angle of 34.82° indicates a position in the 0°–45° range, suggesting that internal strengths exert a somewhat stronger strategic pull than external opportunities. The intensity coefficient of 0.2989 indicates a moderate strategic signal, sufficient to support directional recommendations while acknowledging that the position is not extreme.

The Quadrant I (SO) positioning indicates that the recommended macro-strategic orientation is a growth and development strategy that leverages internal strengths to capitalise on external opportunities, while managing weaknesses and threats as secondary priorities. In practical terms, this suggests Shangluo should prioritise resource-leveraging and opportunity-seizing strategies over defensive approaches.

### Sensitivity analysis results

Table 4 presents the sensitivity analysis results across 12 perturbation scenarios. The strategic quadrant assignment remained in Quadrant I (SO strategy) across all tested scenarios, indicating that the positioning result is robust to moderate variations in dimension-level weights.

**Table 4:** Sensitivity analysis results: strategic positioning under dimension weight perturbations.

Scenario	Perturbation	Δx	Δy	θ (°)	ρ	Quadrant
Base case	None	0.2454	0.1706	34.82	0.2989	I (SO)
S1	S + 10%	0.2820	0.1564	29.02	0.3225	I (SO)
S2	S − 10%	0.2088	0.1849	41.52	0.2789	I (SO)
S3	S + 20%	0.3186	0.1421	24.04	0.3489	I (SO)
S4	S − 20%	0.1722	0.1991	49.14	0.2632	I (SO)
W1	W + 20%	0.2214	0.1762	38.51	0.2830	I (SO)
W2	W − 20%	0.2694	0.1651	31.49	0.3160	I (SO)
O1	O + 10%	0.2316	0.2049	41.49	0.3093	I (SO)
O2	O − 10%	0.2592	0.1363	27.76	0.2929	I (SO)
O3	O + 20%	0.2178	0.2391	47.68	0.3235	I (SO)
T1	T + 20%	0.2524	0.1362	28.35	0.2868	I (SO)
T2	T − 20%	0.2385	0.2050	40.68	0.3146	I (SO)

In each scenario, the perturbed dimension’s weight is adjusted by the indicated percentage, and the remaining three dimensions are rescaled proportionally to maintain a sum of 1.0. θ = azimuth angle; ρ = intensity coefficient. All 12 scenarios maintain Quadrant I (SO) assignment.

**Fig. 3:**
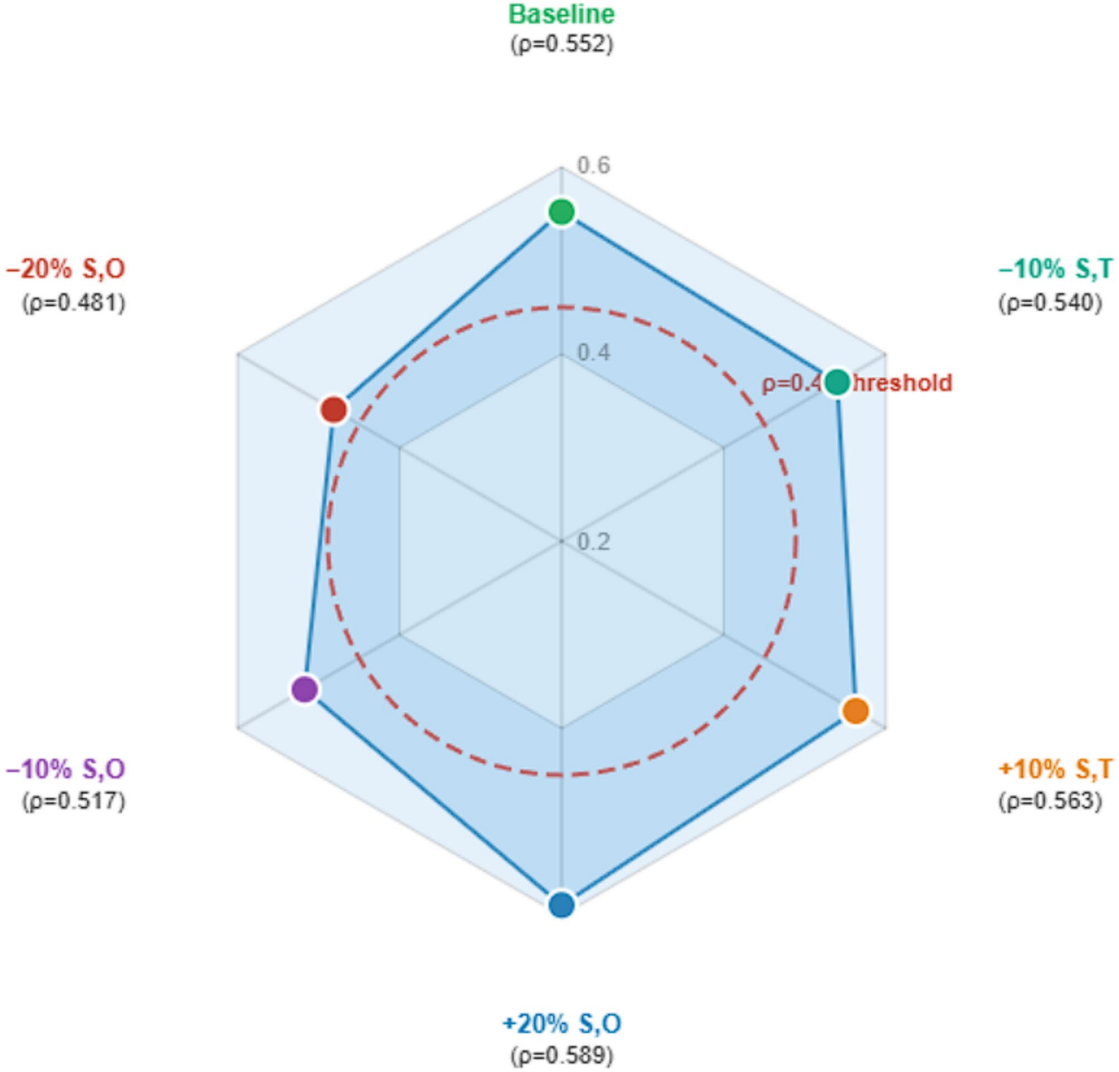
Sensitivity analysis: strategic intensity coefficient (ρ) across scenarios.

While the absolute values of the strategic coordinates and the azimuth angle vary meaningfully across scenarios (θ ranges from 24.04 to 49.14°), the quadrant assignment is invariant. The most challenging scenario for the Quadrant I assignment would be a simultaneous increase in Weaknesses and Threats combined with a decrease in Strengths and Opportunities; however, even the individual worst-case perturbations (S − 20%, W + 20%, O − 10%, T + 20%) maintain positive values for both Δx and Δy with comfortable margins. These results support the conclusion that the SO strategic orientation is robust and not an artefact of marginal weight differences (Fig. 3).

## Discussion

### Interpretation of key findings

The AHP results reveal a strategic landscape in which Shangluo’s forest wellness tourism development is shaped predominantly by its ecological resource endowment, with external policy and market factors providing a reinforcing enabling environment. The dominance of ecological resource quality (S_1_, composite weight 0.1175) as the highest-weighted individual factor across all 18 factors is consistent with broader theoretical arguments that nature-based tourism destinations compete primarily on environmental quality rather than built infrastructure. This finding reflects the expert panel’s recognition that Shangluo’s forest ecosystems—characterised by documented negative air ion concentrations of 3000–12,000 ions/cm^3^, exceptional biodiversity, and favourable climate—constitute the irreplaceable foundation of the region’s competitive proposition.

However, the simultaneous identification of ecological fragility (T_1_) as the highest-weighted threat factor creates a strategic tension that merits careful consideration. Shangluo’s forests are simultaneously its greatest asset and its most vulnerable resource, a duality that has been described as the “conservation-exploitation paradox” in nature-based tourism^55^. The expert panel’s recognition of this tension—assigning top rank to ecological resources among strengths and top rank to ecological fragility among threats—suggests a sophisticated understanding of the development challenge. Translating this recognition into effective management requires institutional mechanisms such as carrying capacity monitoring, adaptive management protocols, and zoning systems that are not yet fully developed in Shangluo.

The relatively high weight assigned to the Strengths dimension overall (0.3652, highest among all four dimensions) warrants careful interpretation. One reading is that Shangluo’s internal resource endowment genuinely constitutes its most powerful strategic asset. An alternative reading—which we consider equally plausible—is that the expert panel, which comprised individuals with professional stakes in the region’s tourism development, may have systematically overweighted positive dimensions relative to an objective assessment^54^. The inter-group comparison (Table 3) provides partial support for this concern: government officials assigned the lowest Weaknesses weight (0.0876) and the highest Strengths weight (0.4012) among all professional categories, which could reflect either informed optimism based on planned investments or institutional reluctance to emphasise deficiencies. This potential for optimistic bias is a recognised limitation of expert-based assessment methods^54^ and should be considered when interpreting the strategic positioning results.

The concentration of weight within the Weaknesses dimension—infrastructure deficits (W_1_) alone accounting for 53.96% of Weaknesses weight—also admits multiple interpretations. The straightforward reading is that infrastructure represents the binding constraint and should receive priority investment. An alternative interpretation is that infrastructure deficits are more salient and easily observable than talent shortages or service quality gaps, which are more diffuse and harder to evaluate. If so, the relatively low weights assigned to talent shortage (W_2_, 0.0296) and service quality (W_3_, 0.0160) may understate their strategic importance. Evidence from mature forest wellness destinations in Japan and South Korea suggests that human capital quality is the primary differentiator of visitor experience once basic infrastructure is in place, indicating that these factors warrant more attention than their current weights might imply^10–12^.

The strong combined weight of the two leading opportunity factors—national/provincial policy support (O_1_, 0.0985) and Xi’an market access (O_2_, 0.0869)—reflects the dual enabling environment that characterises Shangluo’s strategic context. The prominence of government policy factors as an opportunity (O_1_) alongside local government policy attention as a strength (S_3_) reflects the distinctly Chinese institutional reality in which both central and local state capacity co-determine development trajectories^56,57^. This pattern of government-related factors appearing prominently across multiple SWOT dimensions is notably more pronounced than in comparable studies from South Korea or European contexts, consistent with the more directive role of government in Chinese tourism development.

### Theoretical implications

Before interpreting the specific findings, it is important to distinguish between this study’s methodological and conceptual contributions. Methodologically, this study provides a procedurally transparent application of the integrated SWOT-AHP strategic positioning framework to a specific destination context, with detailed documentation that addresses transparency concerns in the extant literature. The conceptual contributions are distinct: they concern what the expert-derived weight patterns reveal about the theoretical relationships between resource endowment, institutional capacity, and competitive positioning in nature-based wellness tourism. The following discussion focuses on these conceptual insights, while acknowledging that all weight-based inferences reflect expert-perceived priorities rather than objective measurements.

The findings speak to three ongoing theoretical conversations in tourism development research. First, regarding the relationship between resource endowment and competitive advantage, our results support a qualified version of the resource-based view as applied to tourism destinations. Shangluo’s ecological and cultural resources clearly constitute the foundation of its perceived strategic position according to the expert panel but the AHP results also demonstrate that resource endowment alone is insufficient: institutional factors—particularly policy support frameworks (O_1_, ranked 2nd) and government policy attention (S_3_, ranked 6th)—received notable composite weights, suggesting that the conversion of natural resources into competitive advantage is mediated by institutional capacity. This finding is consistent with destination competitiveness models that posit “supporting factors” (institutions, infrastructure, human capital) as moderating the relationship between “core resources” (nature, culture, climate) and competitive outcomes^32^.

Second, the study contributes to debates about the applicability of Western-originated planning frameworks in Chinese institutional contexts. The SWOT-AHP framework, originally developed in Western strategic management literature, proved operable in Shangluo, but the factor structure that emerged from the Delphi process reflects distinctly Chinese institutional realities. The prominence of government policy factors across both the Strengths and Opportunities dimensions is consistent with Wade’s developmental state framework^57^, which posits that government coordination overcomes market failures in late-industrialising contexts. Our findings suggest an extension of this logic from its original manufacturing focus to the wellness tourism sector. This does not invalidate the framework but indicates suggests that the relative weighting of institutional versus market factors is context-dependent, a finding that should inform the application of similar frameworks in other state-led development contexts.

Third, and most centrally from a conceptual standpoint, our findings contribute to the literature on forest wellness tourism as a distinct tourism typology.The expert panel’s simultaneous assignment of the highest strength weight to ecological resource quality (S_1_) and the highest threat weight to ecological fragility (T_1_) gives quantitative expression to what may be termed the “conservation-exploitation paradox” inherent in nature-dependent wellness destinations: the very resource that constitutes competitive advantage is also the resource most vulnerable to degradation through tourism activity. This paradox is not merely a methodological artefact but reflects a genuine structural tension that is likely generalisable to other forest wellness destinations globally, and which should be explicitly incorporated into planning frameworks for such destinations.The factor structure validated through the Delphi process—particularly the identification of wellness-specific factors such as negative air ion concentrations, phytoncide exposure potential, and traditional medicinal plant knowledge—supports the argument that forest wellness tourism is not merely a subcategory of ecotourism but a distinct product type with specific resource requirements, value propositions, and market dynamics. The weights assigned to wellness-specific factors (climate comfort, S_5_; wellness industry growth, O_3_; public health awareness, O_4_) relative to generic tourism factors suggest that strategic planning for forest wellness tourism should employ wellness-specific assessment criteria rather than relying on general tourism development frameworks.

### Strategic implications

The following strategic priorities are directed primarily at municipal and county-level government planners responsible for tourism development in Shangluo, while also informing private sector investors, academic institutions, and forest management agencies. In the specific institutional and ecological context of Shangluo City, as assessed by the expert panel,the Quadrant I (SO) strategic positioning, combined with the specific weight distribution across factors, indicates four strategic priorities that are proposed as contextually appropriate considerations forforest wellness tourism development planning. These are presented ascontextually grounded proposals based on expert-perceived weight patterns identified through the AHP analysis. They should not be understood as universally applicable prescriptions or as conclusions that have been empirically validated through demand-side data, ecological measurement, or economic impact assessment.They should be understood as directional guidance rather than prescriptive blueprints, as strategic recommendations derived from expert-based assessments require validation through implementation experience and triangulation with community perspectives and market research.

*Priority* 1: *Ecological resource protection as the non-negotiable foundation*. The convergence of ecological resources as both the top-ranked strength (S_1_, 0.1175) and the source of the top-ranked threat (ecological fragility, T_1_, 0.0549) establishes environmental protection as the precondition for all other strategic actions. A comprehensive ecological carrying capacity assessment system is proposed as an appropriate priority action, including real-time visitor flow monitoring in sensitive areas, seasonal access restrictions for biodiversity-critical zones, and annual ecological health audits conducted by independent assessors. Specific carrying capacity thresholds should be determined through dedicated ecological studies rather than tourism planning exercises alone. This recommendation is consistent with precautionary approaches advocated for protected area tourism management^58^ and with China’s ecological civilisation policy framework^59^.

*Priority* 2:* Phased, wellness-oriented infrastructure investment*. The dominance of infrastructure deficits within the Weaknesses dimension (W_1_, local weight 0.5396) identifies physical infrastructure as the most critical internal constraint. Rather than generic infrastructure expansion, wellness-oriented infrastructure development is proposed that directly supports the forest wellness value proposition in this context: forest therapy trail networks designed to recognised standards, wellness accommodation facilities integrating biophilic design principles, and health monitoring stations at key forest sites. Infrastructure investment should be phased to align with realistic demand projections, and public–private partnership models may be more realistic than purely private financing, particularly in the early phases.

*Priority* 3: *Human capital development as the medium-term differentiator*. Although talent shortage (W_2_, composite weight 0.0296) received a relatively modest weight, evidence from mature forest wellness destinations suggests that human capital quality becomes the primary differentiator of visitor experience as physical infrastructure gaps close. Early investment in professional training programmes is proposed, including partnerships with established forest therapy training institutions for knowledge transfer, establishment of a forest therapy certification programme through Shangluo University, and creation of internship pipelines linking tourism management programmes to local wellness tourism enterprises. This priority is assigned medium-term status because training programmes require institutional development lead time that makes immediate large-scale impact unlikely.It should be noted that the relatively modest AHP weight assigned to talent shortage (W_2_) may reflect the difficulty of quantifying human capital gaps through expert assessment rather than their true strategic importance, as discussed in Section “Interpretation of key findings”.

*Priority* 4: *Leveraging policy momentum and market access for early positioning*. The high combined weight of policy support (O_1_, 0.0985) and market access (O_2_, 0.0869) creates a time-sensitive strategic opportunity. National and provincial policy support for forest wellness tourism is currently strong but may not persist indefinitely as policy priorities evolve. Similarly, the Xi’an source market’s interest in wellness tourism creates a window of opportunity that will narrow as competing destinations develop their own offerings. The development of signature wellness tourism products that establish distinctive brand identity…are all proposed as contextually appropriate strategic actions for the current development phase in Shangluo. Marketing investment should be calibrated to the actual quality of the visitor experience available at each phase to avoid reputational risk from premature market entry.

### Limitations

This study is subject to several methodological and contextual limitations that should be considered when interpreting the results.

First, the reliance on expert judgment introduces inherent subjectivity. Although the Delphi process achieved acceptable consensus (Kendall’s W = 0.76) and AHP consistency ratios were within acceptable bounds, expert-based methods remain vulnerable to shared cognitive biases, including anchoring effects and availability bias. The inter-group weight comparison (Table 3) revealed systematic differences between professional categories, suggesting that panel composition influences results. A different panel—with greater representation of environmental scientists, community residents, or potential visitors—might produce meaningfully different weight assignments. The absence of community voices is a particular limitation, as local residents’ perspectives on development priorities may diverge substantially from those of professional experts^60^.

Second, the SWOT-AHP framework imposes structural constraints that may not fully capture strategic complexity. The framework treats SWOT dimensions as independent categories, whereas in reality, strengths and weaknesses are often interdependent, and the boundary between internal and external factors can be ambiguous (government policy appears in both Strengths as S_3_ and Opportunities as O_1_, reflecting different aspects of the same institutional environment).This fundamental reliance on expert judgment has a critical implication for interpreting the entirety of this study’s findings: all results—including factor weights, strategic coordinates, and quadrant positioning—represent expert-perceived strategic priorities rather than objectively verified development conditions. The study does not directly assess actual tourism demand, verify economic impacts, measure ecological carrying capacity, or document health-related outcomes of forest exposure in Shangluo. It should therefore not be characterised as a strongly empirical study in the conventional sense, but rather as a structured synthesis of expert judgment that provides a systematic, transparent, and reproducible basis for strategic orientation.

Third, the study is cross-sectional, reflecting expert assessments at a single point in time (July–October 2023). The completion of the Xikang High-Speed Railway, for instance, could significantly alter the weight assigned to geographic location (S_4_) and infrastructure deficits (W_1_). Longitudinal application of the framework at regular intervals would provide more dynamic strategic guidance.

Fourth, the generalisability of findings is limited by the single-case design. While Shangluo shares characteristics with many mountain areas in western China, the specific weight distributions and strategic positioning are contextually embedded and should not be directly transferred to other destinations without local adaptation.

Fifth, the study does not incorporate direct demand-side data, and more critically, the perspectives of local residents, host communities, and potential visitors are entirely absent from the analytical framework. All assessments were made by supply-side experts—academics, government officials, industry practitioners, and health professionals—whose professional interests may systematically differ from the lived experiences and development priorities of community members. Forest wellness tourism, as an activity that integrates ecosystem services, health promotion, tourism economics, and local livelihoods, cannot be comprehensively assessed for sustainability through expert judgment alone. Local communities bear the primary costs of tourism-related ecological disturbance, cultural change, and infrastructure development while receiving benefits that may be unevenly distributed. Their absence from this study constitutes a significant gap that substantially limits the study’s capacity to comment on the social and distributional dimensions of sustainable development. The authors acknowledge this as a material limitation rather than a minor methodological note: a complete assessment of forest wellness tourism sustainability requires participatory methods that incorporate community voices. Future research should incorporate visitor surveys and stated preference experiments for demand-side validation, as well as participatory community assessments to capture the social sustainability dimensions that the present expert-panel approach cannot address.

### Future research directions

The limitations identified above suggest several productive directions for future research. First, incorporating demand-side perspectives through visitor surveys and choice experiments would test whether the supply-side priorities identified in this study align with market preferences. Second, longitudinal replication of the SWOT-AHP assessment at two-to-three-year intervals would capture strategic dynamics and enable evaluation of whether recommended interventions have shifted the weight distribution over time. Third, comparative multi-case studies applying identical methods across several Qinling-region destinations would enable direct inter-destination comparison and identification of region-wide versus destination-specific strategic factors. Fourth, integration of quantitative environmental data (carrying capacity measurements, biodiversity indices, air quality monitoring) with expert-based assessments would provide empirical grounding for ecological factors that currently rely on expert judgment. Fifth, qualitative research exploring community residents’ perspectives would address the participatory gap in the current study and contribute to more socially inclusive development planning.

## Conclusions

This study applied an integrated SWOT-AHP strategic positioning framework to assess the development potential of forest wellness tourism in Shangluo City, Shaanxi Province, China. Through a three-round Delphi consultation with 46 experts and AHP pairwise comparisons by 28 experts, 18 validated strategic factors were identified and weighted across four SWOT dimensions. The principal findings are summarised below in direct correspondence with the three research questions posed in Section “Introduction”.

In response to RQ1, which asked what key internal and external factors affect forest wellness tourism development in Shangluo and how they are distributed across the four SWOT dimensions, the three-round Delphi process identified 18 validated factors: 5 Strengths, 4 Weaknesses, 5 Opportunities, and 4 Threats. Strengths centred on ecological resource endowment and traditional wellness culture; Weaknesses were dominated by infrastructure deficits and professional talent shortages; Opportunities reflected favourable policy environments and proximate market demand from the Xi’an metropolitan area; and Threats encompassed ecological fragility, regional competition, market uncertainty, and financing constraints. The progressive improvement in expert consensus across rounds (Kendall’s W rising from 0.52 to 0.76) indicates that the final factor set represents a robust, collectively validated characterisation of the strategic landscape.

Regarding RQ2, which asked how these factors should be prioritised to inform resource allocation, AHP weighting, based on expert panel judgments, indicated that the Strengths dimension received the highest overall weight (0.3652), followed by Opportunities (0.3428), Threats (0.1722), and Weaknesses (0.1198). At the individual factor level, ecological resource quality emerged as the single most influential factor (composite weight 0.1175), followed by policy support frameworks (0.0985), traditional wellness culture (0.0871), and Xi’an market access (0.0869). Notably, infrastructure deficits dominated the Weaknesses dimension, accounting for 53.96% of total Weaknesses weight (composite weight 0.0647), suggesting it as the expert-perceived primary internal constraint. These expert-derived weight patterns suggest that, in the Shangluo context, resource allocation may appropriately prioritise ecological asset protection as the non-negotiable foundation, while directing investment toward infrastructure improvement as the most critical bottleneck to be addressed.

With respect to RQ3, which asked what overall strategic orientation the integrated analysis suggests and how robust this positioning is, the four-quadrant strategic positioning placed Shangluo in Quadrant I (SO strategy) with coordinates P(0.2454, 0.1706), an azimuth angle of 34.82°, and an intensity coefficient of 0.2989. The Quadrant I placement suggests that, from the perspective of the expert panel, a growth-oriented competitive strategy…may represent an appropriate macro-strategic direction for the current development phase. The azimuth angle (34.82°, within the 0–45° range) further suggests that internal strengths exert a somewhat stronger strategic pull than external opportunities. Sensitivity analysis confirmed the robustness of this positioning across all 12 tested scenarios, with θ ranging from 24.04 to 49.14°, ρ from 0.2632 to 0.3489, and Quadrant I maintained throughout, indicating that the strategic recommendation is not an artefact of marginal weight differences.

These findings support four strategic priorities: ecological protection as a non-negotiable foundation, phased wellness-oriented infrastructure investment, medium-term human capital development, and early market positioning to capitalise on current policy momentum. Each recommendation is grounded in specific weight patterns identified through the AHP analysis and is accompanied by implementation considerations and risk assessments.

The study makes three contributions to the forest wellness tourism literature. First, it provides a systematic expert-based assessment of perceived development potential for a specific destination type—mountain forest wellness in western China—where such assessments remain scarce despite rapid policy-driven development. Second, the detailed Delphi-AHP procedural documentation—including factor operational definitions, inter-group weight comparisons, sensitivity analysis, and explicit methodological caveats—offers a replicable template that addresses procedural transparency concerns raised in the SWOT-AHP literature. Third, the identification of the conservation-exploitation paradox (ecological resources simultaneously constituting the top-weighted strength and the source of the top-weighted threat) highlights a strategic tension likely generalisable to other nature-dependent wellness tourism destinations and requiring explicit institutional attention in development planning.This paradox—quantitatively expressed through the simultaneous top-ranking of ecological resource quality as a strength and ecological fragility as a threat—constitutes the study’s most theoretically generalisable conceptual contribution, as it identifies a structural tension inherent to any nature-dependent wellness destination and not merely an artefact of local conditions.

We conclude with a note of methodological humility. Readers should interpret the findings of this study as reflecting expert-perceived strategic priorities at a single point in time, subject to the cognitive biases and professional perspectives of the participating panel. The quantitative precision of the AHP outputs conveys methodological rigour in processing expert judgments, but does not transform those judgments into objective measurements of development conditions. The study’s value lies in providing a transparent, reproducible, and structured basis for strategic conversation—not in prescribing development outcomes. Its value lies in providing a systematic, transparent, and replicable basis for strategic orientation—identifying where to focus attention and resources—rather than in prescribing specific development actions.

## Data Availability

The data used to support the findings of this study are available from the corresponding author upon request.
